# Sir Roy Calne (1930–2024): Tribute to a Founding Father of ELITA, Honouring a Pioneer in Liver and Intestinal Transplantation

**DOI:** 10.3389/ti.2024.12811

**Published:** 2024-02-23

**Authors:** Hermien Hartog, Giacomo Germani, Renè Adam, The European Liver and Intestinal Transplant Association (ELITA), a section of ESOT

**Affiliations:** ^1^ Department of Surgery, University Medical Center Groningen, Groningen, Netherlands; ^2^ Multivisceral Transplant Unit, Department of Surgery, Oncology and Gastroenterology, Padua University Hospital, Padua, Italy; ^3^ Centre Hepato-Biliaire AP-HP, Hôpital Paul Brousse, Université Paris-Saclay, Villejuif, France

**Keywords:** liver transplantation, intestinal transplantation, organ transplantation, history, obituary

It is with great sadness and sympathy that we pay tribute to one of the founding fathers of liver and intestinal transplantation in Europe. As the passing of Sir Roy Calne, may he rest in peace, was announced, an era came to an end of great innovations and the introduction of organ transplantation. We stand on the shoulders of giants, of those who pioneered immunosuppression fearlessly, performed courageous surgery and had the grit and foresight to establish formidable international networks to progress the field of transplantation.

One of the decisive moments for Sir Roy Calne was when, as a young professional, he saw a patient of his own age with end-stage organ failure. He pursued the possibility of organ transplantation and was appointed Professor of Surgery in Cambridge at age 35, recognizing the immense potential of the new immunological discoveries in the early 60 s. His pioneering work on Cyclosporin, Campath and kidney transplantation is history.

Short in stature and with an amiable expression, Sir Roy Calne described himself as a “somewhat rebellious” character [1]. He stood the tide by performing many “firsts”; firsts that were previously dismissed as impossible. He was the first in Europe to prove that patients could survive following a liver transplant. He pioneered intestinal transplantation in the United Kingdom. And took part in the first teams to perform clusters of transplants of heart, liver and lung, and multivisceral plus kidney. He unlocked and believed in the enormous potential that organ transplantation could provide for patients who would otherwise die from organ failure.

Sir Roy Calne also acknowledged the limitations of his profession and more universally, of the human race. In interviews, Sir Roy Calne comes across as someone profoundly touched by the paradoxes of life. While the gift of transplantation gave many individual people suffering from lethal disease and their families a new leash of life, he did not leave unmentioned ethical problems in living or deceased organ donation that can occur when power is abused. Being described as a wonderful father to his six children, he examined the problem of world population density in his book “Too many people” [2, 3]. As a surgeon and artist with paintings exhibited in the Science Museum in London, he displayed an outstanding ability to combine candour, humanity, art, and critical thinking [4].

On October 25, 1993, the founding meeting for the European Liver Transplant Association (later ELITA) took place in Rhodes. This meeting was set up by the then Chairs of the ESOT Steering Committee, Professor Jean Bernard Otte and Professor Sir Roy Calne. Eight years prior to this milestone, in 1985, Sir Roy Calne, alongside Professors Henri Bismuth and Rudolph Pichlmayr, initiated the European Liver Transplant Registry (ELTR) in Munich [5]. This registry was established to document all liver transplant procedures across Europe and fostered a collaborative scientific community among European liver transplant centers. We are deeply grateful to Sir Roy Calne for this legacy to establish a successful association of professionals in Europe progressing the potential of liver and intestinal transplantation, which continues until today. We honour his tremendous contributions to science, surgery and medicine in organ transplantation.

Our thoughts are with his wife and children, family and friends.

## Data Availability

The original contributions presented in the study are included in the article/supplementary material, further inquiries can be directed to the corresponding author.

## Group members of The ELITA Board

Constantino Fondevila, Deniz Balci, Eliano Bonaccorsi, Giacomo Germani, Hermien Hartog, Luca Belli, Miriam Cortes Cortisuelo, Pal-Dag Line, Rene Adam, Sarwa Darwish Murad, Silvio Nadalin, Thierry Artzner.
